# Items

**Published:** 1879-04

**Authors:** 


### Items.

COMMENCEMENT EXERCISES OF RUSH MEDICAL COLLEGE.— The thirty-sixth annual Commencement exercises of Rush Medical College took place in the presence of a large audience in the Third Presbyterian Church of this city on the afternoon of the 25th of February. Out of one hundred and thirty-eight who entered the graduating class, one hundred and twenty-three received the degree of doctor of medicine. That so large a number failed to pass the examinations is no discredit to the gentlemen themselves, but we take it as an evidence that the professional attainments demanded by the college are higher than ever before, and as a consequence the class which has just graduated, taken as a whole, is just so much the better qualified to enter upon the duties of their profession.

The exercises were opened with prayer by the Rev. Theo. N. Morrison, Jr., after which followed the conferring of degrees; the charge of the President; response on behalf of the class by Dr. Charles E. Fogg, and the valedictory address by Dr. A. B. Strong. Following the valedictory address was the presentation by the class of a complete case of surgical instruments to Dr. Strong. Subjoined are the names of the graduates:

Chauncy Willard Amy, Marion J. Anderson, Erastus Yoemans Arnold, Samuel Bailey, Clarence Perley Battles, Rufus Henry Bartlett, Edwin Julius Bartlett, A.M., Robert Wesley Baker, Osron Dorcelia Benson, Stillman Marion Benner, Benjamin Jephthah Bill, William Thomas Bishop, Adelbert Henry Bowman, William Burgess Brengle, John Franklin Bradshaw, Charles Theodore Burchard, Martin Caldwell, Charles David Camp, James Cavaney, George Gillette Chittenden, William Wallace Cole, A.B., Albert Stewart Core, Charles John Creighton, Willis Edward Crane, Theodore Parker Crosse, Stephen Cummings, Charles Eustache Cyrier, John Oscar Dawson, Edward Davies,

James Blaney Devlin, Constantine Lomax Dicken, James Michael Dinnen, George Warritte Dosh, Cyrus Donaldson, Julian Arthur Dubois, Thaddeus Augustus Dumont, Karl Friedrick Wilhelm Eberlein, James Plaster English, Heman Ephram Farnsworth, Charles Elwin Fogg, A.B., Henry Jacob Fleischer, Thomas Benton Francis, Otto Tiger Freer, William Henry Harrison Gable, Benjamin Marvin Gill, Orris William Grant, Thomas Baldwin Graham, Bernard Charles Gudden, Addison Hawkins, Edward Leander Hills, William Wesley Hitchcock, Charles Henry Holmes, Elwyn Ashworth Holroyd, Harry Pettit Huntsinger, Henry Porter Johnson, Francis Marion Jordan, Samuel L. Kilmer, Charles Krusemarck, Antonio Lagorio, William Henry Lanyon, Fred Willard Lester, James Lonsdale, James Ancel Lord; Edward Macdonald, George Lemuel Marshall, Allan Aleyne Mathews, Edgar Jehial Meacham, William Meyer, Charles Frederick McComb, Hugh E. McCaw, John Wilkinson McCausland, John Calhoun McClintock, Charles William McGavren, Carroll Everard Miller, William Emil Julian Michelet, Albert Roscoe Mitchell, John Vincent Moran, Daniel Grove Moore, Harold Nicholas Moyer, Timothy Douglas Murphy, John Benjamin Murphy, Joseph Aloysius Muenich, John Tenbrook Newton, John Francis O'Keefe, John Walter O'Connor, Harlow N. Orton, William Enos Parker, Emery Allen Paschal, John Thompson Rice, Charles Winter Robbins, Charles Alexander Rogers, M.D., Joseph Louis Ross, Moses Archie Rush, B.S., Rockwood Sager, Ora Owen Sawyer, B.S., William Raymond Shinn, John Campbell Sheridan, Anton Shimonek, Courtney Smith, George Lewis Smith, William Theodore Frelinghuysen Smith, William Peter Smith, Francis Marion Smiley, Thomas Jefferson Sprague, Jr., Theodore Parker Stanton, Simon Strausser, George Charles Stockman, B.S., James Harrison Stipp, Charles Stuart, M.D., John H. Thornton, William Porter Verity, William Philander Walker, Solon Robertson Wakefield, James Wallace, Francis Alvin Weir, Florado Houser Wellcome, Frederick Charles Werner, PH.G., Herman L. Wilson, M.D., David Henry Worthington, Frank Rubin Woodard, Magnus Youngstedt. Honorary, Dr. J. H. Gardiner.

At half-past seven in the evening a largely attended reunion of the alumni of the college took place at the Tremont House. This was the first union which has occurred since the disastrous fire of 1871, which not only destroyed the college and its records, but also the records of the Association, so that the addresses of its scattered members were lost, the constitution of the Association was burned and amid the general wreck none had found time to take the necessary steps toward reorganization. Recently Dr. J. H. Etheridge succeeded in obtaining the addresses of over twenty-two hundred of the alumni; a committee was appointed to discover the names of the old officers, and make the necessary preparations for the reunion. Invitations to a banquet were extended by the faculty of the college to all of the alumni of the college.

A partial copy of the old constitution had been found, and from it a new one was drafted, which was adopted. The President of the Association, Dr. C. Goodbrake, of Clinton, Illinois, who was elected in 1870, had been found, and he was present to preside at the reorganization. The officers elected for the ensuing year were: President, Dr. Robt. M. McArthur; 1st Vice President, Dr. Wm. Fox; 2nd Vice President, Dr. Harold N. Moyer; Secretary and Treasurer, Dr. E. Fletcher Ingals. Executive Committee, Drs. Norman Bridge, V. L. Hurlbut and F. A. Emmons.

After the business of reorganization had been accomplished, the retiring President delivered an able address which received the hearty endorsement of the members; after which the gentlemen adjourned to the banquet hall.

After the banquet the regular toasts were offered and responded to in a very happy manner.

Dr. Abner Hard in answer to the toast "Our Elder Brothers," gave a brief history of the early days of Alma Mater; Dr. E. W. Lee spoke for the County Hospital Staff; Dr. J. E. Owens gave a history of the spring faculty; Dr. Fox spoke in feeling terms for the alumni scattered throughout the length and breadth of the land; Dr. S. L. Kilmer spoke for the class of '79; Dr. V. L. Hurlbut responded to the toast "An old 'Rush,'" and Prof. J. Adams Allen for Alma Mater. The regular toasts being concluded, Dr. Goodbrake again took the chair and called for the unfinished business, under which head Dr. E. Ingals introduced the following resolutions, which were ordered published, and at his request laid over until the next annual meeting for discussion.

The Alumni Association of the Rush Medical College desire to express in a formal and public manner the affectionate regard which they entertain for their Alma Mater, their pride in her past history, and their confident hopes for her increased usefulness in the future. We hold that her supreme duty to the public, which has always accorded her a generous support, demands that her first aim and object should be to aid in axalting the attainments of the medical profession, and by so doing increasing its usefulness, power and respectability. As a mode of accomplishing these results we would respectfully offer to the Board of Trustees of the College, the following suggestions:

*First.*—To increase the regular annual term of college instruction to a period of not less than nine months.

*Second.*—To require attendance on three full terms of medical lectures as a pre-requisite to admission to examination for the degree of Doctor of Medicine.

*Third.*—To institute preliminary examinations for students who apply for matriculation to the school, admitting only such as have at least a thorough English education.

The miscellaneous business was followed by impromptu speeches from several gentlemen. Many instructive and witty remarks were made which we are unable to publish. Dr. Etheridge suggested that a portion of the annual dues be appropriated for prizes, for the encouragement of original investigation. Dr. Gunn stated that the Faculty of the college considered, and should at all future reunions consider the association as their guests, and should therefore insist upon defraying all the expenses of the banquet.

Adjourned to meet again commencement eve, 1880.

THE following new chairs have been established by the Trustees and Faculty of Rush Medical College, and filled by appointments of the gentlemen named:

A professorship of Gynæcology—Dr. Wm. H. Byford.

A professorship of Orthopædic Surgery—Dr. Jno. E. Owens.

A professorship of Dermatology—Dr. James Nevins Hyde.

TWENTIETH COMMENCEMENT EXERCISES OF THE CHICAGO MEDICAL COLLEGE.—MEDICAL DEPARTMENT OF NORTHWESTERN UNIVERSITY.

The twentieth annual Commencement of this college was held in the Plymouth Church, on the afternoon of March 4th, 1879. The church was well filled with an interested audience. The platform, which was tastefully decorated with flowers, was occupied by the Faculty and some of the Trustees of the University, and the front central pews by the members of the graduating class. Prayer was offered by the Rev. Dr. Everest. Certificates for the undergraduates, and for hospital and dispensary service, were distributed; and the degree of Doctor of Medicine was conferred upon the members of the graduating class by Prof. N. S. Davis, Dean of the Faculty, aided by the recording secretary, Prof. L. Curtis. A very appropriate valedictary address was delivered by Prof. S. J. Jones, and an unusually excellent response in behalf of the class, by F. E. Stevens, M.D. The first prize for the best inaugural thesis was awarded to Charles Herbert Fegers, whose thesis contained an excellent account of *A New Spinal Supporter*, devised by himself. The second prize was awarded to Henry Clay Mitchell, for a thesis on the *Peristaltic Action or Motion of the Mesenteric Blood-vessels.* At suitable intervals throughout the exercises, excellent music was given by Benj. Owen, the organist and by the Blaney Quartette. The following are the names of the graduating class: John Francis Abel, Burton B. Aplington, Charles Henry Bryant, Ed. Livingston Cary, Lorents Andreas Claussen, Shobel Vail Clevenger, Francis Jewell Crane, Charles Herber Fegers, Adalbert R. Fellows, William Griggs Goffe, Omar Oakley Hall, Everett Charles Hartley, Dennis John Hayes, Ernest Clark Helm, William Malcolm Jackson, Hugh Lawrence Jenks, Homer L. Leland, Thomas Smith McDavitt, Matthew Harrison McKillip, Henry Clay Mitchell, William David Morgan, John Francis Mulholland, Penn Walker Ransom, William Henry Roberts, George William Robinson, William Henry Schick, Smith Augustus Spilman, Frank Eugene Stevens, John Stout, Norton Strong, Abraham Lukert Thomas, Robert VanDeusen, John M. Wilcox, William Calvert Wolf, Ansel Woodworth, George Harvey Wright. To these were added the names of Robert H. Brown and Henry W. Waldeman, examined at the close of the first term of the college year; and Philemon D. Harding, of Goshen, Indiana, on whom was conferred the Honorary Degree of M.D., making *thirty-eight* regular degrees out of a total class of students numbering one hundred and fifty-six.

ALUMNI ASSOCIATION:

The thirteenth annual re-union of the Alumni Association of the Chicago Medical College was held at the Palmer House in the evening of the same day. An unusually large number of members of the association were present, and the proceedings were of an interesting and important character, Dr. N. Senn, of Milwaukee, presided. A brief address of welcome was delivered by Dr. Blanchard, in behalf of the committee of arrangements, and the proceedings of the last meeting were read by the secretary, Dr. D. A. K. Steele. A large number of new members were initiated, and a very interesting necrological report was read by Prof. M. P. Hatfield. The president, Dr. N. Senn, delivered a very interesting and valuable address on the importance of the thorough study of anatomy. The Association took some important action, not only earnestly endorsing the long and graded course of instruction established by the college, but affording substantial aid in adding to the appliances and usefulness of the physiological laboratory. After the business of the Association had been transacted, an elegant banquet was enjoyed in the usual manner until a late hour.

ANNUAL MEETING OF ILLINOIS STATE MEDICAL SOCIETY.—The regular annual meeting of the Illinois State Medical Society will be held at Lincoln, Illinois, commencing at 10 o'clock, a. m., of Tuesday, May 20th, 1879, and continue two or three days. The local committee of arrangements will make all proper efforts to render the meeting pleasant and profitable to all who may attend. The following standing and special committees are expected to report:

*Committee of Arrangements.*—Drs. R. P. Wilson, C. T. Wilbur, L. L. Leeds, P. L. Diefenbacker and N. S. Reed.

*Committee on Practical Medicine.*—Drs. G. W. Jones, J. T. Crow and L. B. Moore.

*Committee on Surgery.*—Drs. John E. Owens, H. H. Deming and H. Z. Gill.

*Committee on Obstetrics.*—Drs. C. C. Hunt, David S. Booth and W. M. Kaul.

*Committee on Gynæcology.*—Drs. T. D. Fitch, P. L. Diefenbacker and N. Bridge.

*Committee on Ophthalmology and Otology.*—Drs. S. J. Jones, J. Perrin Johnson and H. B. Young.

*Committee on Drugs and Medicines.*—Drs. J. F. Todd, C. B. Johnson and John Wright.

*Committee on Necrology.*—Drs. T. F. Worrell, G. W. Albin and E. Ingals.

*Committee on Syphilis.*—Dr. W. M. Chambers.

*Committee on Psychological Medicine.*—Dr. A. McFarland.

*Committee on the Advancement of Physical Science.*—Dr. S. H. Stevenson.

*Committee on Croup.*—Dr. H. Z. Gill.

*Committee on Use of Hydrate of Chloral and Bromide of Ammonium in Obstet. Practice.*—Dr. C. H. Norris.

*Committee on Medical Education.*—Drs. E. Ingals, R. G. Bogue and D. Prince.

Each permanently organized local medical society is entitled to one delegate for every five of its resident members; the faculty of each regular medical college is entitled to two delegates; and the staff of every chartered or municipal hospital to one delegate. It is hoped that the profession of the entire State will be well represented at the coming meeting.

N. S. DAVIS, *per. Secretary.*

CHICAGO, ILL., March 15th, 1879.

A LETTER has been received by the Illinois State Board of Health from Göttingen, Germany, asking whether there is such a medical school as the University of Medicine and Surgery of Haddenfield, New Jersey. It seems that a diploma of this institution was presented by a veterinary surgeon in Berlin, who was trying to get a German degree, but owing to the fact that German universities have a rule that a degree from a small one is to be held equal, and therefore not to be superceded by a degree from a large institution, this university refused to grant another degree. A copy of the *Göttinger Zeitung* was also received, containing a copy of the diploma, and, as was supposed by Prof. Schwarz, it proves to be fraudulent, as there is no school of this character at Haddenfield. The letter also states that there is an agency in Germany for the sale of spurious diplomas.

VITAL STATISTICS.—CHICAGO, March 15, 1879. *Dear Sir:* At the last meeting of the West Chicago Medical Society, the resolution given below was discussed and adopted with great unanimity. We hope that you will take an early opportunity to secure its consideration by your local or county medical society, and also by the State Medical Society.

NORMAN BRIDGE, M.D., *Prest. West Chicago Med. Soc'y.*
W. T. BELFIELD, M.D., *Secretary.*

*Resolved*, That in the opinion of the West Chicago Medical Society, it is the duty of the State Board of Health to procure an amendment of the law relating to the collection of Vital Statistics, securing the incorporation of those sections of the New Hampshire and Connecticut laws which provide for the compensation of persons who make returns of births and deaths, at the rate of twenty-five cents for each birth or death returned and recorded within the limits of the State.

THE sudden death of Dr. Jno. M. Woodworth, late Supervising Surgeon General of the United States Marine Hospital Service, is already known to many of our readers, from the general publicity which the event has occasioned. Dr. Woodworth, as is well known, was a physician of this city, and his professional associates and friends in Chicago have felt, even more keenly than others, the loss his death has occasioned to the roll of honor in the profession of this city, as well as to the public service.

We propose to publish, at an early date, a fit memorial of his life and character.

